# Analysis of Candidate Idarubicin Drug Resistance Genes in MOLT-3 Cells Using Exome Nuclear DNA

**DOI:** 10.3390/genes9080390

**Published:** 2018-08-01

**Authors:** Tomoyoshi Komiyama, Atsushi Ogura, Takehito Kajiwara, Yoshinori Okada, Hiroyuki Kobayashi

**Affiliations:** 1 Department of Clinical Pharmacology, Tokai University School of Medicine, Kanagawa 259-1193, Japan; 2 Nagahama Institute of Bio-Science and Technology, Shiga 526-0829, Japan; a_ogura@nagahama-I-bio.ac.jp (A.O.); b312009@m.nagahama-I-bio.ac.jp (T.Ka.); 3 Support Center for Medical Research and Education, Tokai University, Kanagawa 259-1193, Japan; yosi@is.icc.u-tokai.ac.jp

**Keywords:** leukemia, MOLT-3, gene polymorphism, genetic variations, idarubicin, drug resistance

## Abstract

Various gene alterations related to acute leukemia are reported to be involved in drug resistance. We investigated idarubicin (IDR) resistance using exome nuclear DNA analyses of the human acute leukemia cell line MOLT-3 and the derived IDR-resistant cell line MOLT-3/IDR. We detected mutations in MOLT-3/IDR and MOLT-3 using both Genome Analysis Toolkit (GATK) and SnpEff program. We found 8839 genes with specific mutations in MOLT-3/IDR and 1162 genes with accompanying amino acid mutations. The 1162 genes were identified by exome analysis of polymerase-related genes using Kyoto Encyclopedia of Genes and Genomes (KEGG) and, among these, we identified genes with amino acid changes. In resistant strains, *LIG* and helicase plurality genes showed amino-acid-related changes. An amino acid mutation was also confirmed in polymerase-associated genes. Gene ontology (GO) enrichment testing was performed, and lipid-related genes were selected from the results. Fluorescent activated cell sorting (FACS) was used to determine whether IDR permeability was significantly different in MOLT-3/IDR and MOLT-3. The results showed that an IDR concentration of 0.5 μg/mL resulted in slow permeability in MOLT-3/IDR. This slow IDR permeability may be due to the effects of amino acid changes in polymerase- and lipid-associated genes.

## 1. Introduction

We aimed to characterize the molecular mechanisms underlying idarubicin (IDR) resistance in acute leukemia cells. It is known that gene alterations in acute leukemia cells are involved in drug resistance. A better understanding of the mechanisms underlying drug resistance in these cells will help to improve the effectiveness of chemotherapy. In this study, we investigated the mechanisms underlying drug resistance in the human acute leukemia cell line MOLT-3 and its IDR-resistant derivative MOLT-3/IDR by complete nuclear DNA analyses.

In recent years, the mortality rate of leukemia has been gradually decreasing due to increased bone marrow transplantations; development of antibiotic, antifungal, and antiviral drugs; optimization of transfusion therapy; analysis of treatment protocols by multicenter studies; and development of molecularly targeted drugs [1,2]. In general, IDR, an anthracycline antitumor agent, is used as a therapeutic agent for acute myelogenous leukemia. IDR has also been used for acute myeloid leukemia, blast crisis of chronic myelogenous leukemia, and recurrence of acute lymphocytic leukemia. IDR has a superior remission induction effect than daunorubicin, and remission induction therapy using idarubicin was reported to be effective in clinical trials of recurrent acute lymphocytic leukemia [3]. IDR acts by inhibiting the transcription of RNA from DNA, which in turn prevents the growth of cancer cells. Moreover, IDR is highly lipophilic and can therefore maintain a high intracellular drug concentration even in P-glycoprotein-expressing cells. However, antitumor drug resistance in solid and recurring tumors has decreased the effectiveness of antitumor drugs [1,2,4]. As patients with leukemia often respond differently to treatment, IDR resistance is becoming an increasing problem and a significant barrier in treating patients. Therefore, understanding of the IDR resistance mechanism will be useful for the development of molecular targeted drugs in the future.

Since 1995, we have conducted many experiments using the MOLT-3 cell line to determine the genetic cause of IDR resistance. We have previously created cultures of various tumor cell lines that are resistant to many antitumor drugs, including anthracycline [5,6,7,8]. We also analyzed the molecular mechanisms underlying resistance in these cells to identify mutations or abnormal expression of drug resistance genes [9,10,11,12,13,14,15,16,17]. We have shown that it is possible to overcome drug resistance by using nucleic acid formations to suppress the expression of the genes that contribute to drug resistance. Abnormal activation of kinase pathways has been observed in a range of malignant tumors, as well as in some resistant cells. Furthermore, it has been confirmed that it is possible to overcome resistant cells by suppressing small interfering RNA (siRNA) or nucleic acid preparation in kinase pathways [15]. Analysis of the biomarkers associated with drug response may be useful for treating cancer. We previously analyzed these biomarkers using cell panels treated with antitumor agents and opioid analgesics, which are major obstacles in managing cancer treatment [18,19,20].

Recently, we identified putative drug resistance genes using comparative genomic hybridization (CGH) array and whole mitochondrial DNA sequence analyses [21]. We identified a unique mutation site (p.Thr61Ile) in the *ND3* gene of mitochondrial DNA in the MOLT-3/IDR cell line. From CGH array analysis, we extracted five candidate drug resistance genes and focused specifically on the *GALNT2* gene involved in O-linked glycosylation of lipids. A mutation in the stop codon of *GALNT2* leads to 18 additional amino acids being translated in the mutated protein compared with the wild-type. However, we could not obtain a detailed evaluation at the single-nucleotide polymorphism (SNP) level or mutated amino acid level. Our aim is to develop a method to overcome drug resistance by combining nucleic acid formulation and inhibitors that target important resistance factors.

To understand the cause of drug resistance, we investigated the underlying mechanisms using nuclear DNA analyses of the exomes of MOLT-3 and MOLT-3/IDR cells. In addition, we attempted to identify mutations in MOLT-3/IDR and MOLT-3 cells by using both Genome Analysis Toolkit (GATK) and SnpEff program [22]. Exome analysis is a method of comprehensive analysis using only exon sequences, which is used in gene resistance analyses. For example, some researchers have induced the disappearance of SNPs and the emergence of new SNPs, suggesting that the emergence of drug-resistant clones from patient genes is possible [23,24,25,26,27,28,29,30,31].

In the future, we aim to target certain expression factors and gene mutations in resistant cells to identify possible causes of differential cancer gene expression, independent of normal cells.

## 2. Materials and Methods

### 2.1. Generation of MOLT-3/IDR Cell Lines

To study the mechanism underlying IDR resistance, an IDR-resistant cell line was established from the human acute leukemia cell line MOLT-3 (American Type Culture Collection (ATCC), Manassas, VA, USA) [18]. MOLT-3 cells were treated with 40 nM IDR (Idamycin, Pfizer, New York, NY, USA) for 8 h, at which point the surviving cells were subcultured weekly with IDR. The concentration of IDR was increased by 20 nM at each exposure [32]. After 4 months, IDR resistance was tested by 3-(4,5-dimethylthiazol-2-yl)-2,5-diphenyltetrazolium bromide (MTT) assay, and the cells were found to be 10 times more resistant to IDR. These cells were designated MOLT-3/IDR (Table S1) [21]. Single colonies were subcultured and retested by MTT assay before cryopreservation. After thawing, the cells were cultured for 1 month before use in subsequent experiments.

Similar attempts were undertaken to establish IDR-resistant subclones from the cell lines K562 and CCRF-CEM(CEM) to overcome concerns regarding instability in the MOLT3 system. However, increasing the resistance by 10-fold in these cell lines was difficult.

### 2.2. Cell Lines and Culture Conditions

MOLT-3 and MOLT-3/IDR (ATCC) cells were cultured in Roswell Park Memorial Institute (RPMI) 1640 Medium (Nacalai Tesque, Kyoto, Japan) containing penicillin (50 IU/mL) and streptomycin (50 µg/mL) and supplemented with 10% fetal calf serum. MOLT-3 and MOLT-3/IDR cells were cultured for 2 days at a density of 1.6 × 10^6^ cells/8 mL RPMI1640 culture medium per 100 cc cell culture dish (Eppendorf, Hamburg, Germany).

### 2.3. Exome Analysis of MOLT-3 and MOLT-3/IDR

We performed exome analysis to investigate the IDR resistance mechanism in MOLT-3 and MOLT-3/IDR cells. Exomes of DNA samples were enriched using previously captured platforms obtained using the SureSelect XT Reagent Kit and SureSelect XT Human All Exon V5 (Agilent Technologies, Santa Clara, CA, USA) and assembled using the SeqNovaCS Data Analysis System at Hokkaido System Science Co., Ltd. (Sapporo, Japan). Prepared libraries were then sequenced with 2 × 100 bp paired-end reads on Hiseq1000 and HiSeq 2500 sequencers (Illumina, San Diego, CA, USA).

For mutational analysis, we used the GATK tool [33]. The Unified Genotyper GATK tool was used to identify mutant genotypes in both MOLT-3 and MOLT-3/IDR. Using hg38 obtained from the University of California (Santa Cruz, CA, USA) as a reference, we identified mutation sites within the genotypes of each sample. In addition, we annotated our findings in SnpEff [22]. For the identified mutations, we confirmed the information already available in the database, such as the presence or absence of the gene or amino acid mutations. Based on information obtained from the SnpEff [34] annotation, we compared the mutations detected in MOLT-3 and MOLT-3/IDR cells and identified genes with mutations accompanied by specific amino acid mutations in MOLT-3/IDR cells. We created a script for the extraction of these genes, and then investigated the genes that were assumed to be related to IDR resistance mechanisms.

### 2.4. KEGG Pathway Analysis

IDR has been shown to inhibit nuclear polymerase activity, potentially by cutting one or both strands of double-stranded DNA before inhibiting recombination by the enzyme topoisomerase II. Therefore, we performed Kyoto Encyclopedia of Genes and Genomes (KEGG) pathway analysis to reveal the functions of polymerase-related genes and other specific mutated genes [35]. The selected pathways were used as DNA replication pathways for complex network analysis of proteins and enzymes that play a role in and are required for DNA replication.

### 2.5. GO Enrichment Test

Identified genes were used to perform the gene ontology (GO) enrichment test using the gene ontology enrichment analysis and visualization tool (GOrilla) [36]. The GOrilla enrichment test allocates an Ensembl ID to each gene based on the information about gene function provided by the GO test. Within a set of detected genes, common functions become apparent by studying gene relationships. This system allowed us to visualize gene relationships by comparing many functions within a set of detected genes. The reference was set to *Homo sapiens*, and enrichment tests were performed using the single ranked list of genes. The *p*-value threshold was set at 10^−3^.

### 2.6. Drug Treatment

IDR was dissolved in an Otsuka distilled water solution (Otsuka, Osaka, Japan). After 2 days of culturing, MOLT-3 and MOLT-3/IDR cells were suspended in RPMI 1640 culture medium in a 24-well culture plate (Eppendorf) with the density adjusted to 2 × 10^5^ cells/mL/well. MOLT-3 and MOLT-3/IDR cells were cultured without (control) or with IDR (0.1, 0.5, and 1 mg/mL) for 10 min.

### 2.7. Flow Cytometric Analysis

Concentration experiments were conducted according to the method described by Smeets et al. [32]. IDR was used at concentrations of 0, 1, 10, and 50 µg/mL; it was added to the cells and cells were washed within 60 min. In the preliminary experiment, cells were recovered by trypsinization; 1 mL of the medium containing cells was centrifuged; the supernatant was separated using a pipette; 1 mL PBS was added; the mixture was then centrifuged and aspirated; further, 300 µl PBS was added; and lastly, the mixture was subjected to fluorescent activated cell sorting (FACS) analysis. This experiment was repeated twice. Based on the results, we selected a concentration of 1 µg/mL, which compared between the MOLT 3 cells and IDR cells. Because of this reaction, we performed another detailed test with the three concentrations 0, 0.1, and 0.5 µg/mL, with one set for 30 min and the other set for 10 min. After IDR treatment, the cells were recovered by trypsinization; 1 mL of the medium containing treated cells was subjected to centrifugation; the supernatant was aspirated; 1 mL medium was again added, and the mixture was centrifuged; the supernatant was removed, 300 µL medium was added; and lastly, the mixture was subjected to FACS analysis. In addition, time from the point of washing was also considered. Measurements were done at 0, 30, and 90 min; in the intermittent period, the mixture was incubated in a CO_2_ incubator. After 10 min of incubation, the cells were washed twice with RPMI 1640 culture medium and added to 300 µL of RPMI 1640 culture medium in 5 mL Falcon round-bottom polystyrene tubes (CONING, New York, NY, USA). IDR quantification was performed using a BD Fortessa cytometry analyzer (Beckton Dickinson, San Jose, CA, USA) and analyzed with FlowJo version 7.6.5 software (TreeStar, San Carlos, CA, USA).

## 3. Results

### 3.1. Detection of Mutations Using GATK and SnpEff

We detected mutations by comparing the exome sequence data (MOLT-3 and MOLT-3/IDR) with the reference genome using GATK. For this, we did not map MOLT-3 and MOLT-3/IDR directly when attempting to identify the mutations, as both MOLT-3 and MOLT-3/IDR cells contain fragmented short-leads.

**Figure 1:**
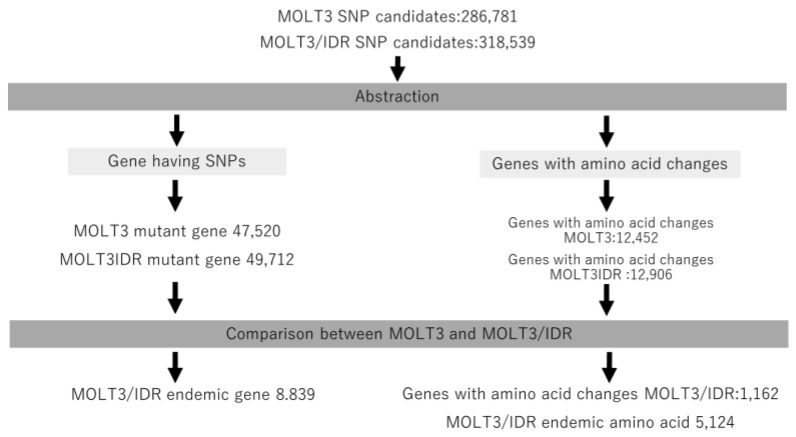
Procedure for identifying specific gene mutations. We extracted mutated genes and genes with amino acid changes from both MOLT-3 and MOLT-3/IDR cells. In addition, we extracted specific genes that only existed in MOLT-3/IDR cells and specific genes with amino acid changes, and then determined the number of mutations in specific amino acids by comparing them. **SNP**: Single Nucleotide Polymorphism.

We then annotated the detected mutations using SnpEff and compared the mutations detected in MOLT3 and MOLT-3/IDR cells according to the annotated information. This procedure is shown in Figure 1. Further, we extracted the mutated genes and genes with amino acid changes from both MOLT-3 and MOLT-3/IDR cells. We also extracted specific genes only found in MOLT-3/IDR and specific genes with amino acid changes, and determined the number of mutations in specific amino acid sequences by comparison. The results are summarized in Table 1 and are labeled as mutations in MOLT-3, mutations in IDR-MOLT3, and number of specific mutations in MOLT-3/IDR. Specific mutations in MOLT-3/IDR were obtained by comparing MOLT-3 and MOLT-3/IDR, and extracting the mutations that occur specifically in MOLT-3/IDR. The total number of mutations increased with the number of genes in the category, including splicing variants, noncoding genes, and predicted genes. Consequently, we identified 8839 genes with specific mutations and 1162 genes with accompanying amino acid mutations in MOLT-3/IDR cells (Tables S2 and S3). In addition, 5124 mutated amino acids were found to exist specifically in MOLT-3/IDR cells.

### 3.2. Results of KEGG Pathway Analysis

In addition to inhibiting nuclear polymerase activity, IDR cuts DNA strands by inhibiting the enzyme topoisomerase II, which recombines DNA by cutting one or both strands of double-stranded DNA. Using this mechanism, we examined mutations of polymerase-related genes and identified 1162 genes from exome analysis, which were then investigated by KEGG pathway analysis. We then identified genes containing the expected amino acid changes (Table S2).

**Figure 2:**
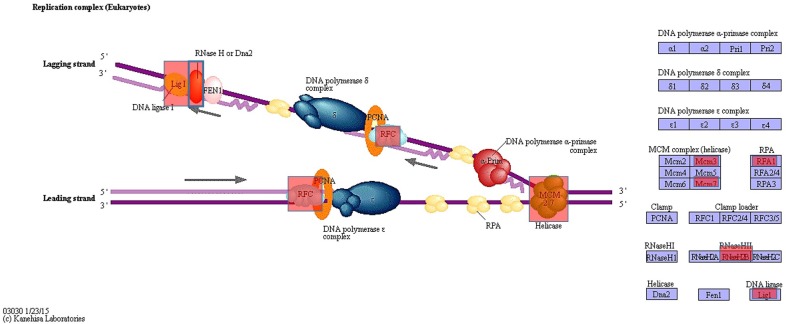
Polymerase-related genes identified using Kyoto Encyclopedia of Genes and Genomes (KEGG) pathway analysis. DNA replication of genes related to protein functions and genes related to transcription are shown. Circles indicate genes with amino acid changes. We did not observe any amino acid changes or gene mutations in topoisomerase genes. However, it is possible that common mutations were removed in MOLT-3/IDR and MOLT-3 cells. Adapted from [35].

Figure 2 shows the classification of the identified genes into the three groups: DNA replication genes, genes related to protein functions, and genes related to transcription. Circles indicate genes with amino acid changes. We did not observe any amino acid changes or gene mutations related to topoisomerase. However, it is possible that common mutations were removed among MOLT-3/IDR and MOLT-3 cells. For these mutations, we analyzed amino acid changes in the domain using the Uniprot database. Mutations were identified in polymerase-related genes associated with the observed amino acid changes. However, we did not detect mutations in any domain. Nevertheless, given the effect of IDR, we can consider that provided the mutations were found in genes, IDR is somehow involved (Table 2).

### 3.3. Analysis of Genes Using the Gene Ontology Enrichment Test

One aim of this study was to determine the most common functions in a set of 1162 genes identified by exome analysis. To achieve this, GOrilla was used to analyze biological processes and cell structure/molecular functions [37]. Further, in vivo processes related to gene function were visualized based on the annotation of each gene. mutations were removed in MOLT-3/IDR and MOLT-3 cells.

**Figure 3:**
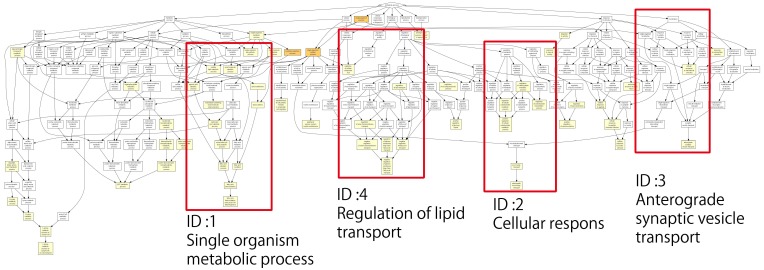
Functional analysis using the Gene Ontology (GO) enrichment test. The related processes and relationships of a number of identified genes are shown. The lower the *p*-value, the larger the difference in expression level compared with the reference. *p*-values are color coded as follows: white, > 10^−3^; light orange, 10^−3^ to 10^−5^; orange, 10^−5^ to 10^−7^; dark orange, 10^−7^ to 10^−9^; red, < 10^−9^. The next graph was created by considering the process with the lowest *p*-value from the lower layer of each extracted process and then displaying it in a graph. The ID shows its location in Figure 3. ID4 mainly represents the removed processes that relate to fatty acids, and also one process that was removed regardless of its *p*-value as an example.

Figure 3 shows the relationships and related processes of a single identified gene; the lower the *p*-value, the larger the difference in expression level compared with the reference. The *p*-values are color-coded as described in the figure legend. The second graph was created by considering the processes with the lowest *p*-values from the lower layer of each extracted process and then displaying it as a graph. The ID shows its location in Figure 3. ID4 mainly represents removed processes that are related to fatty acids, as well as one process that was removed regardless of the *p*-value as an example. This was not used in the analysis. Of the processes removed, five were involved in the metabolism of fatty acids (Table 3).

According to the Cancer Chemotherapy Center [38], newly synthesized fatty acids thrive despite containing numerous exogenous lipids, and a number of lipid metabolism enzymes, including fatty acid synthase, contribute to cancer development and transformation. In particular, the fatty acid metabolism enzyme acyl-COA synthetase acts as an inhibitory agent in the mitochondria-dependent intrinsic apoptotic pathway. This has been found to impact cancer survival rates and resistance to drugs. Therefore, it is believed that variations in lipid-related genes that include fatty acids may be related to IDR tolerance levels. To confirm this, a GO enrichment test was performed, which included the previously removed processes and lipid-related genes selected from the results. The selected genes are shown in Table 4. Uniprot was used to determine whether domain amino acids changed in the removed genes. We found that domain amino acids were altered in adiponectin (ADIPOQ), arachidonate 5-lipoxygenase (ALOX5), and ALOX15 genes. The C1 domain in ADIPOQ and lipoxygenase domain amino acids in ALOX5 and ALOX15 were found to be altered. ADIPOQ is related to lipid oxidation, fatty acid oxidation, fatty acid catabolism, lipid modification, and lipid metabolism [39,40,41,42,43,44,45,46,47,48]. ALOX5 is also related to lipid metabolism [49,50,51,52,53]. ADIPOQ, ALOX5, and ALOX15 are reportedly associated with lung cancer, colorectal cancer, and colon cancer [54,55,56,57,58]. In particular, *ALOX5* has attracted increasing attention as a key gene for drug targeting and overcoming drug resistance in patients with leukemia [59,60,61,62,63,64,65,66,67].

Mutations associated with amino acids in lipid-related genes and mutations in the domains of amino-acid-related genes were also present.

### 3.4. Analysis of IDR Permeability in MOLT-3/IDR and MOLT-3 Cells by FACS

**Figure 4:**
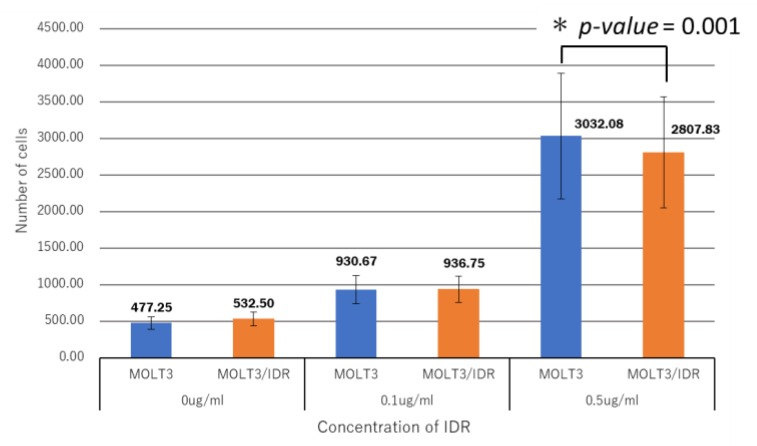
Analysis of idarubicin (IDR) permeability at concentrations of 0, 0.1, and 0.5 μg/mL IDR in MOLT-3/IDR and MOLT-3 cells by fluorescent assisted cell sorting (FACS). Immediately after administration, the MOLT-3/IDR cells treated with 0.5 μg/mL IDR showed slow IDR permeability.

**Figure 5:**
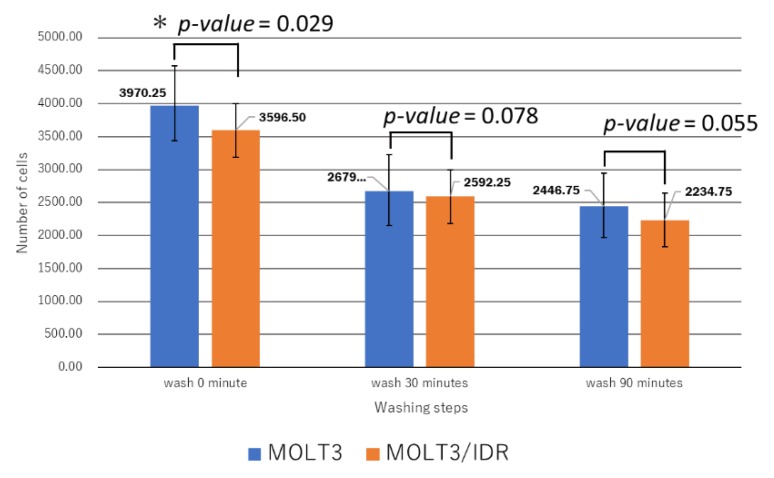
Analysis of IDR permeability according to differences in administration time in MOLT-3/IDR and MOLT-3 cells by FACS. After 0, 30, and 90 min incubation of MOLT-3/IDR cells with 0.5 μg/mL IDR, the permeability was slightly slower. In particular, IDR permeability at 0 min after washing was the slowest in MOLT-3/IDR cells (*t*-test, *p*-value = 0.029; *p*-value < 0.05, *t*-test).

**Figure 6:**
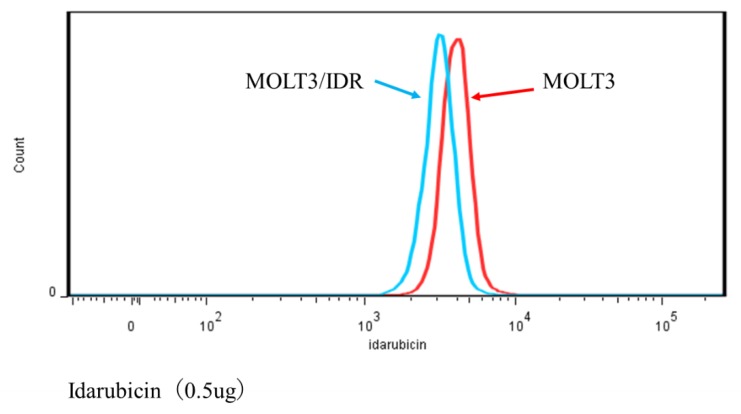
Histogram plots of IDR permeability at a concentration of 0.5 μg/mL in MOLT-3/IDR and MOLT-3 cells.

Numerous mutations in lipids and plasma membrane proteins were detected by the GO enrichment test. Subsequently, FACS was used to study IDR permeability in MOLT-3/IDR and MOLT-3 cells (Table S4). IDR was used at concentrations of 0, 0.1, and 0.5 μg/mL for 0, 30, and 90 min after the washing step. IDR was administered after incubation for 10 min. The measurements were recorded 12 times in total over 2 days, with the results shown in Figure 4, Figure 5, and Figure 6. An IDR concentration of 0.5 μg/mL was found to significantly slow the drug’s permeability (*p*-value = 0.001) in MOLT-3/IDR cells compared with that in MOLT-3 cells. Furthermore, IDR permeability was also slow in MOLT-3/IDR cells at 0 min after washing (*p*-value = 0.029; *p*-value < 0.05). Our results confirmed that an IDR concentration of 0.5 μg/mL resulted in the slowest permeability in MOLT-3/IDR cells immediately after administration at 0 min after washing. After incubation for 30 and 90 min, cells treated with 0.5 μg/mL IDR showed slightly slow permeability. This difference in permeability is believed to be related with IDR resistance. In addition, the fluorescence intensity of IDR permeability of the MOLT-3 cells at a concentration of 0.5 μg/mL was higher than that of the MOLT-3/IDR cells.

## 4. Discussion

In the current study, we investigated the molecular mechanisms underlying IDR resistance using the human T cell leukemia cell line MOLT-3 and its IDR-resistant derivative MOLT-3/IDR. We attempted to identify genes specifically mutated in MOLT-3/IDR cells by both mitochondrial and nuclear DNA analyses.

GATK mutation detection and annotation using SnpEff helped us identify genes associated with amino acid changes. Given the active mechanism of IDR, it was hypothesized that polymerase-related genes would also contain mutations. KEGG pathway analysis showed that *LIG* and helicase plurality genes contained amino-acid-related alterations in resistant strains. Furthermore, amino acid mutations were also found in polymerase-associated genes, but not in the domains. This suggests that while gene function remained unchanged, IDR active sites in the genes were altered, preventing the drug from functioning normally. This may be related to the development of IDR resistance.

Next, a GO enrichment test was performed using GOrilla, in which genes with mutated amino acids were linked to GO terms and the biological processes related to each gene were determined. We found that a large number of genes were related to fatty acid and lipid metabolism. In cancer cells, de novo synthesis of fatty acids thrives regardless of exogenous lipid levels. Through fatty acid synthesis, enzymes associated with acyl-CoA synthetase, lipid metabolism, and fatty acid metabolism promote cancer development and growth. Given their relationship with cancer survival and drug resistance, the detected genes were examined further. We found a large number of genes associated with amino acid mutations in the domain. In addition, mutations resulting in amino acid changes were found in polymerase- and lipid-associated genes.

FACS was then used to determine whether IDR permeability differed significantly between MOLT-3/IDR and MOLT-3 cells, with the results showing that an IDR concentration of 0.5 μg/mL resulted in slow IDR permeability in MOLT-3/IDR cells. In addition, the fluorescence intensity of IDR permeability in the MOLT-3/IDR cells at a concentration of 0.5 μg/mL was lower than that in MOLT cells. This is considered to be a factor indicating resistance. Kapli et al. addressed this by showing that sensitive cells accumulated more drug and showed at least 2-fold greater levels of brightness than the resistant cells [68]. This is believed to be due to the effects of amino acid changes in polymerase- and lipid-associated genes. Whether this is indeed related to the acquisition of IDR resistance will be a topic of a future study; however, the discovery of a number of genes likely related to IDR resistance implies that significant progress has been made in leukemia research in relation to the understanding of the mechanisms behind the acquisition of IDR resistance.

Further studies should focus on the mechanism underlying IDR resistance acquisition to determine whether it is related to amino acid changes. Furthermore, we believe that using the K 562 and CEM cell line, we would like to confirm the results obtained in our analyses the same way. We aim to undertake such research and provide new information on the acquisition of IDR resistance.

The results of the current study will broaden our knowledge on the genetic diversity of the mechanisms associated with drug resistance and genomic and gene correlation research. Moreover, we believe that our research will contribute to next-generation genetics. The use of new bioinformatics technology will form the basis of next-generation cancer genetics research.

## 5. Conclusions

We detected mutations in MOLT-3/IDR and MOLT-3 cells using both GATK and SnpEff. Subsequently, we identified 8839 genes with specific mutations in MOLT-3/IDR cells and 1162 genes accompanied with amino acid mutations. Among the 1162 genes, genes related to polymerases, fatty acid synthesis, and lipid metabolism showed nonsynonymous mutations, suggesting that these genes are related to IDR permeability. FACS was used to determine whether the permeability of IDR was significantly different between MOLT-3/IDR and MOLT-3 cells. The results showed that although there was no significant difference between the two cells, an IDR concentration of 0.5 μg/mL resulted in slow IDR permeability in MOLT-3/IDR cells. This may be due to the effects of amino acid changes found in polymerase- and lipid-associated genes. Taken together, our findings suggest that multiple mutations in the genes identified in the current study are involved in IDR resistance. In the future, we intend to analyze the roles of these genes in IDR resistance in leukemia cells in greater depth by using targeted inhibitors, cell proliferation, and IDR sensitivity assays.

## Figures and Tables

**Table 1:** Genome Analysis Toolkit (GATK) and SnpEff mutant analysis.

	MOLT-3	MOLT-3/IDR	MOLT-3/IDR only
Mutant base	286,781	318,539	194,683
Mutant genes	47,520	49,712	8839
Genes containing amino acid changes	12,452	12,906	1162
Endemic amino acids	33,712	36,489	5124

GATK: Genome Analysis Toolkit; SnpEff: SNP effect; IDR: idarubicin

**Table 2:** Genes with polymerase-related amino acid changes.

Gene	Mutant Amino Acid	Domain	Known or Unknown
LIG1	p.Lys702Glu	×	×
MCM3	p.Ile3Leu	×	×
	p.Asp391Gly	MCM	×
	p.Ala620Pro	×	×
MCM7	p.Val273Ile	×	×
RFC1	p.Gly416Asp	BRCT	×
	p.Leu612Pro	×	×
RNASEH2B	p.Phe95Cys	×	×
	p.Ala287Ser	×	×

BRCT: Breast cancer 1 C-terminal

**Table 3:** GO enrichment test results.

ID	Description	*p*-Value	Number of Genes
1	fatty acid beta-oxidation	1.63 × 10^−5^	3
2	regulation of cellular carbohydrate metabolic process	4.08 × 10^−5^	5
1	lipid oxidation	4.22 × 10^−5^	3
1	fatty acid oxidation	4.22 × 10^−5^	3
1	fatty acid catabolic process	4.22 × 10^−5^	3
1	fatty acid beta-oxidation using acyl-CoA dehydrogenase	5.28 × 10^−5^	2
3	response to tumor necrosis factor	6.70 × 10^−5^	6
4	negative regulation of plasma membrane long-chain fatty acid transport	5.28 × 10^−4^	2

**Table 4:** Lipid-related genes.

Gene	Mutant Amino Acid	Domain	Known or Unknown
*ACOX2*	p.Lys66Thr	×	○
*ACAD10*	p.Arg69Cys	×	×
*ADIPOQ*	p.Tyr216His	c1q	×
*AGK*	p.Thr2Met	×	×
*ALOX15*	p.Asn237Ser	Lipoxygenase	×
*ALDH3A2*	p.Leu479Arg	×	×
*AGPAT9*	p.Glu321Gln	×	×
*ALOX5*	p.Asp465Val	Lipoxygenase	×
*ABHD3*	p.Asp291Tyr	×	×

## Supplementary Materials

The following are available online at http://www.mdpi.com/2073-4425/9/8/390/s1. Table S1: Levels of IDR resistance in MOLT-3 and MOLT-3/IDR cells. Table S2: Genes specific to MOLT-3/IDR cells (8839 genes). Table S3: Genes with amino acid mutations specific to MOLT-3/IDR cells (1162 genes). Table S4: Raw data for IDR Permeability in MOLT-3 and MOLT-3/IDR cells using FACS.
